# Percutaneous coronary intervention using drug-eluting stents versus coronary artery bypass graft surgery in left main coronary artery disease an updated meta-analysis of randomized clinical trials

**DOI:** 10.18632/oncotarget.20142

**Published:** 2017-08-10

**Authors:** Lei Gao, Yuqi Liu, Zhijun Sun, Yabin Wang, Feng Cao, Yundai Chen

**Affiliations:** ^1^ Department of Cardiology, PLA General Hospital, Beijing 100853, China

**Keywords:** left main coronary artery, coronary artery bypass graft, percutaneous coronary intervention, drug-eluting stent, meta-analysis

## Abstract

**Objectives:**

To compare the safety and efficacy of percutaneous coronary intervention (PCI) using drug-eluting stent (DES) and coronary artery bypass graft (CABG) for the treatment of left main coronary artery (LMCA) disease.

**Background:**

Several new randomized trials have recently examined the clinical outcomes of PCI and CABG in LMCA disease. However, the results of these studies were inconsistent.

**Materials and Methods:**

We searched five online electronic databases to identify all the randomized clinical trials assessing the outcomes of PCI using DES and CABG in patients with LMCA. The clinical outcomes were the major adverse cardiac and cerebrovascular event (MACCE), all-cause death, myocardial infarction (MI), stroke, and repeat revascularization (RR).

**Results:**

A total of 5 randomized clinical trials with 4595 LMCA patients were included in this meta-analysis. For one year follow-up, the results indicated that PCI were associated with a lower risk of stroke (RR = 0.21, 95% CI = 0.07–0.65, *P* = 0.007), a higher risk of RR (RR = 1.72, 95% CI = 1.28–2.33, *P* < 0.001) than CABG. Moreover, for long-term follow-up, there were significant higher risks of MACCE and RR with PCI versus CABG (MACCE: HR = 1.26, 95% CI = 1.11–1.44, *P =* 0.001; RR: HR = 1.70, 95% CI = 1.42–2.05, *P* < 0.001). However, there were no significant differences between the two groups in all-cause death and MI risks, regardless of follow-up duration.

**Conclusions:**

PCI is noninferior to CABG in short term follow-up of patients with LMCA disease, but CABG is more safety and efficacy than PCI using DES in long-term follow-up.

## INTRODUCTION

Left main coronary artery (LMCA) disease, one of obstructive coronary artery diseases, is diagnosed in 5–7% of patients who have been referred for coronary angiography [1, 2]. As the extent of ischemic myocardium, patients with significant LMCA disease have poor clinical outcomes and 50% three-year mortality [3]. Increasing numbers of clinical trials have demonstrated that coronary artery bypass grafting (CABG) is beneficial for patient survival and quality of life when compared with optimal medical therapy [4, 5]. As the safety and durability of surgery, current international guidelines have recommended CABG as the reference standard for the treatment of LMCA disease [6, 7].

Percutaneous coronary intervention (PCI) with stenting, a Class II alternative method of LMCA treatment by current guidelined recommend, is becoming more used and shows favorable clinical outcomes for patients with significant LMCA disease that have a high risk of surgery and low or intermediated SYNTAX scores because of many remarkable improvements in medical device technology and adjunctive pharmacotherapies have been achieved during the last decade [8–11]. For example, PCI with drug-eluting stents (DES) using could significantly reduce the risk of clinical restenosis [12, 13]. Recently, randomized controlled clinical trials have compared the safety and efficacy of PCI using DES and CABG for the treatment of LMCA disease [14–21]. One recent meta-analysis included randomized clinical trials to study the 1-year outcomes of PCI using DES versus CABG for patients with LMCA and found that PCI using DES was associated with a lower risk of stroke and a higher risk of repeat revascularization compared with CABG [22].

However, this meta-analysis did not include the latest and the largest study and did not study the long-term outcomes of PCI using DES versus CABG. As a matter of fact, the results of long-term outcomes of PCI using DES versus CABG in these studies were inconclusive. For example, the NOBLE trail found CABG was better than PCI using DES with respect to Kaplan-Meier 5 year estimates of MACCE and revascularisation for treatment of LMCA disease [18]. However, the EXCEL trail demonstrated that PCI using DES was noninferior to CABG in patients with LMCA disease [19].

Therefore, we performed an updated meta-analysis to systematically clarify whether PCI using DES was noninferior on the safety and efficacy to CABG for treatment of LMCA disease during 1-year and long-term period.

## MATERIALS AND METHODS

### Literature search

We searched five online electronic databases, including PubMed and the Excerpta Medica Database (EMBASE), ISI Web of Science, Chinese Biomedical Literature (CBM) database and Chinese National Knowledge Infrastructure (CNKI) database, for all publications examining the safety and efficacy of PCI using DES compared with CABG for the treatment of LMCA disease that had been published through December 31, 2016. The search strategy was based on combinations of the following terms: “coronary artery disease”, “left main”, “percutaneous coronary intervention or PCI”, “drug-eluting stent or DES”, and “coronary artery bypass”. To retrieve the most eligible studies, we manually screened all relevant publications and their reference lists. We included published papers on relevant studies involving human subjects, with no restrictions regarding publication language.

### Inclusion and exclusion criteria

The following eligible studies were included in the meta-analysis: (1) randomized controlled clinical trials; (2) studies whose population of interest included LMCA patients; and (3) studies making comparisons of clinical outcomes between PCIs using DES and CABG; and (4) studies whose clinical outcomes included major adverse cardiac and cerebrovascular event (MACCE), all-cause death, myocardial infarction (MI), stroke, and repeat revascularization (RR). The following studies were excluded from the analysis: (1) duplicates of previous publications; (2) abstracts, reviews, commentaries and editorials; (3) animal studies; and (4) studies without sufficient available original data, even after we had contacted their corresponding authors.

### Clinical outcomes and definitions

The clinical outcomes of this study included MACCE, all-cause death, MI, stroke, and RR. MACCE were defined as the composite of death, stroke, MI, or RR. All-cause death was defined as death from any cause. MI was defined as the documentation of a new abnormal Q-wave after the index revascularization. Stroke was defined as the rapid or sudden onset of new neurological deficit persisting for > 24 hours with no apparent nonvascular cause. RR was defined as ischemia-driven revascularization by either PCI or CAGB. The short term was defined as 1 year, and the long term was defined as > 3 year in this meta-analysis.

### Data extraction

Two reviewers independently extracted the following information from the eligible trails using a standardized data collection form: the trail’s name, period of the procedures, location, ethnicities of the patients, numbers of patients, follow-up duration, clinical outcomes, characteristics of the subjects (age, gender, and underlying diseases, such as hypertension, hyperlipidemia, and diabetes mellitus), clinical characteristics of the patients (previous MI, LVEF (left ventricular ejection fraction), numbers of diseased vessels, EuroSCORE, SYNTAX score, distal LMCA disease, and DES type) in the PCI using DES and CABG groups, and clinical outcomes data. Any disagreements regarding the data extracted by the two reviewers were resolved by discussion until a consensus was reached among all the authors.

### Statistical analysis

The data regarding the one-year outcomes were categorical, and pooled risk ratios (RRs) and their corresponding 95% confidence intervals (CIs) were performed for one-year outcomes’ summary statistics. All studies reported the Kaplan-Meier long-term estimates, and pooled hazard ratios (HRs) and their 95% CIs were conducted for long-term outcomes’ summary statistics. The chi-square-based Cochran Q test and *I*^2^ statistic were employed to assess between-study heterogeneity [23, 24]. Sensitivity analyses were conducted to assess differences by DES type (early- versus newer-generation DES) for long-term outcomes. Funnel plots, Begg’s rank test, and Egger’s linear regression test were performed to examine potential publication bias [25]. *P* value was two sided, and < 0.05 was considered significant. All statistical analyses were conducted using STATA statistical software (version 11.0; Stata Corp, College Station, TX, USA).

## RESULTS

### Characteristics of the included studies

**Figure 1:**
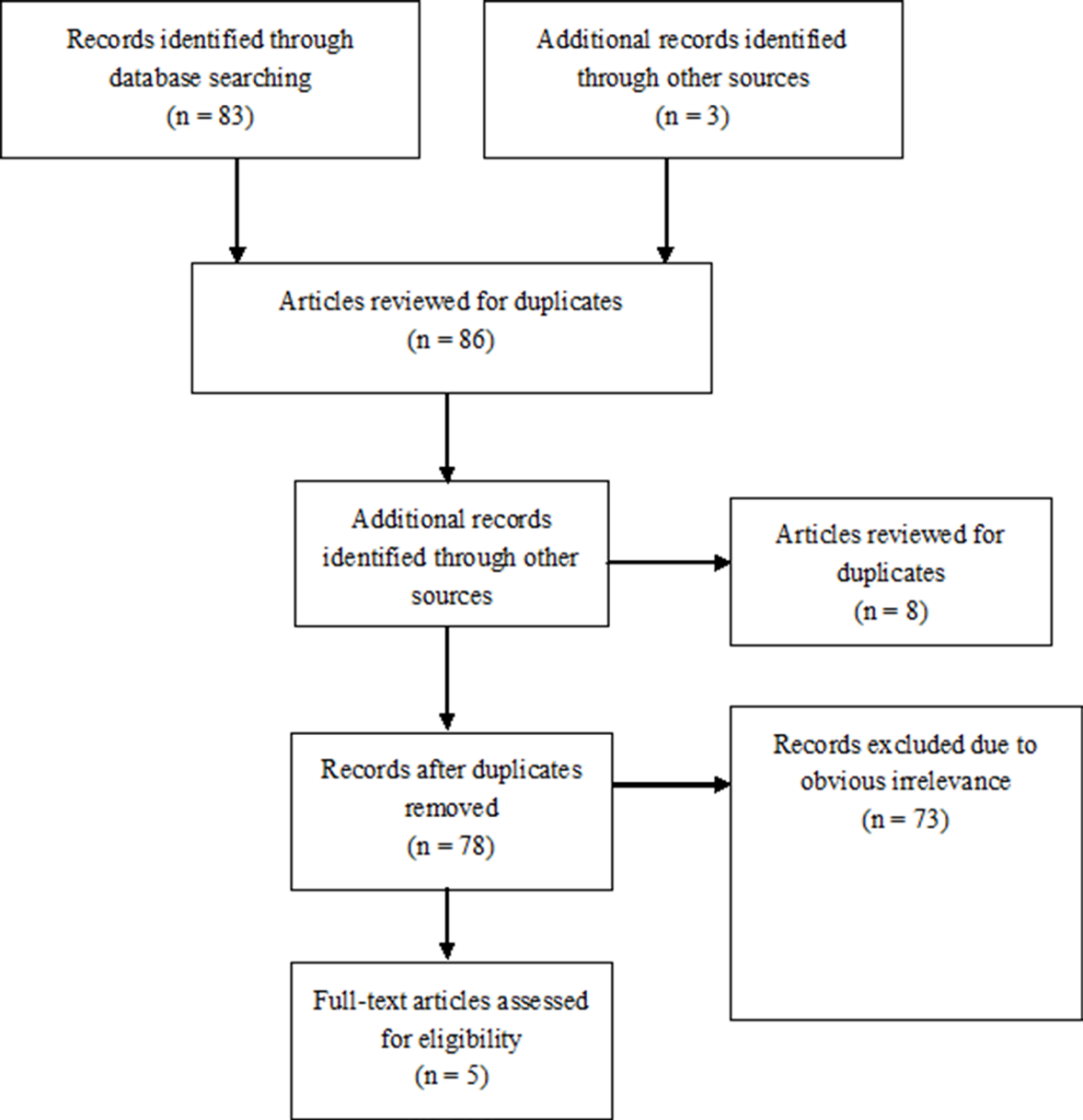
Flow diagram of the study selection process

The flow chart in Figure 1 displays information pertaining to the comprehensive literature search for and selection of studies assessing the safety and efficacy of PCI using DES compared with CABG for the treatment of LMCA disease. As a result of our careful search and study selection process, 5 randomized clinical trials with 4595 LMCA patients, including 2297 patients undergoing PCI using DES and 2298 patients undergoing CABG, were included in our meta-analysis. SYNTAX trial (Synergy Between Percutaneous Coronary Intervention With TAXUS and Cardiac Surgery) was a prospective, multinational, randomized trial, which was designed to assess the optimal revascularization strategy between PCI and CABG for patients with left main coronary disease. PRECOMBAT (Bypass Surgery Versus Angioplasty Using Sirolimus Eluting Stent in Patients With Left Main Coronary Artery Disease) study was a prospective, open-label, randomized trial conducted at 13 sites in South Korea, and was designed to determine the outcomes of PCI compared with CABG for the treatment of unprotected left main coronary artery stenosis. EXCEL (Effectiveness of left Main Revascularization) trial was an international, open-label, multicenter randomized trial that compared everolimus-eluting stents with CABG in patients with left main coronary artery disease. NOBLE (The Nordic-Baltic-British left main revascularization study) study was a prospective, randomized, open-label, non-inferiority trial, done at 36 hospitals in northern Europe, and was to compare PCI and CABG for treatment of left main coronary artery disease. The baseline clinical and procedural characteristics of each trial are summarized in Table 1 and Table 2. Among the 5 trails, 4 were from Western countries and 1 from Asia. Regarding duration of follow-up, 4 studies reported the data of 1-year follow-up and 4 trials had 5-year follow-up clinical data. The prevalence of previous MI ranged from 4% to 36%, with distal LMCA disease ranged from 52% to 81%. The mean EuroSCORE ranged from 2.0% to 3.9%, and the mean SYNTAX score ranged from 22 to 30. Regarding DES type, two trials used early-generation DES in PCI and two trials used newer-generation DES in PCI.

**Table 1:** Characteristics of eligible studies enrolled in the meta-analysis

Trial	Period	Location	Ethnicity	Number of patients (PCI/CABG)	Follow-up duration	Outcomes
Boudriot et al.	2003–2009	Germany	Caucasian	201 (100/101)	1-year	MACCE/Death/MI/RR
PRECOMBAT	2004–2009	South Korea	Asian	600 (300/300)	1-year and 5-year	MACCE/Death/MI/Stroke/RR
SYNTAX	2005–2007	Europe and United States	Caucasian	705 (357/348)	1-year and 5-year	MACCE/Death/MI/Stroke/RR
NOBLE	2008–2015	Europe	Caucasian	1184 (592/592)	1-year and 5-year	MACCE/Death/MI/Stroke/RR
EXCEL	2010–2016	United States	Caucasian	1905 (948/957)	3-year	Death/MI/Stroke/RR

**Table 2:** Patients and procedural characteristics of the included studies

Variable	Boudriot et al.	PRECOMBAT	SYNTAX	NOBLE	EXCEL
Age, y	66/69	62/63	65/66	66/66	66/66
Male, %	72/77	76/77	72/76	80/76	76/78
Diabetes mellitus, %	40/33	34/30	24/26	15/15	30/28
Hypertension, %	82/82	54/51	67/62	65/66	75/74
Hyperlipidemia, %	68/64	42/40	81/75	82/78	72/69
Previous MI, %	19/14	4/7	29/25	NR	18/17
LVEF, %	65/65	62/61	NR	60/60	57/57
No. of diseased vessels, %					
0	28/29	9/11	12/14	NR	17/18
1	35/27	17/18	19/20	NR	31/31
2	26/28	34/30	31/31	NR	35/32
3	11/17	41/41	38/35	NR	17/19
EuroSCORE, %	2.4/2.6	2.6/2.8	3.9/3.9	2/2	NR
SYNTAX score, mean	24/23	24/26	30/30	23/22	21/21
Distal LMCA disease, %	74/69	67/62	56/52	81/81	NR
DES type	SES	SES	PES	BES	EES

### One-year outcomes

**Figure 2:**
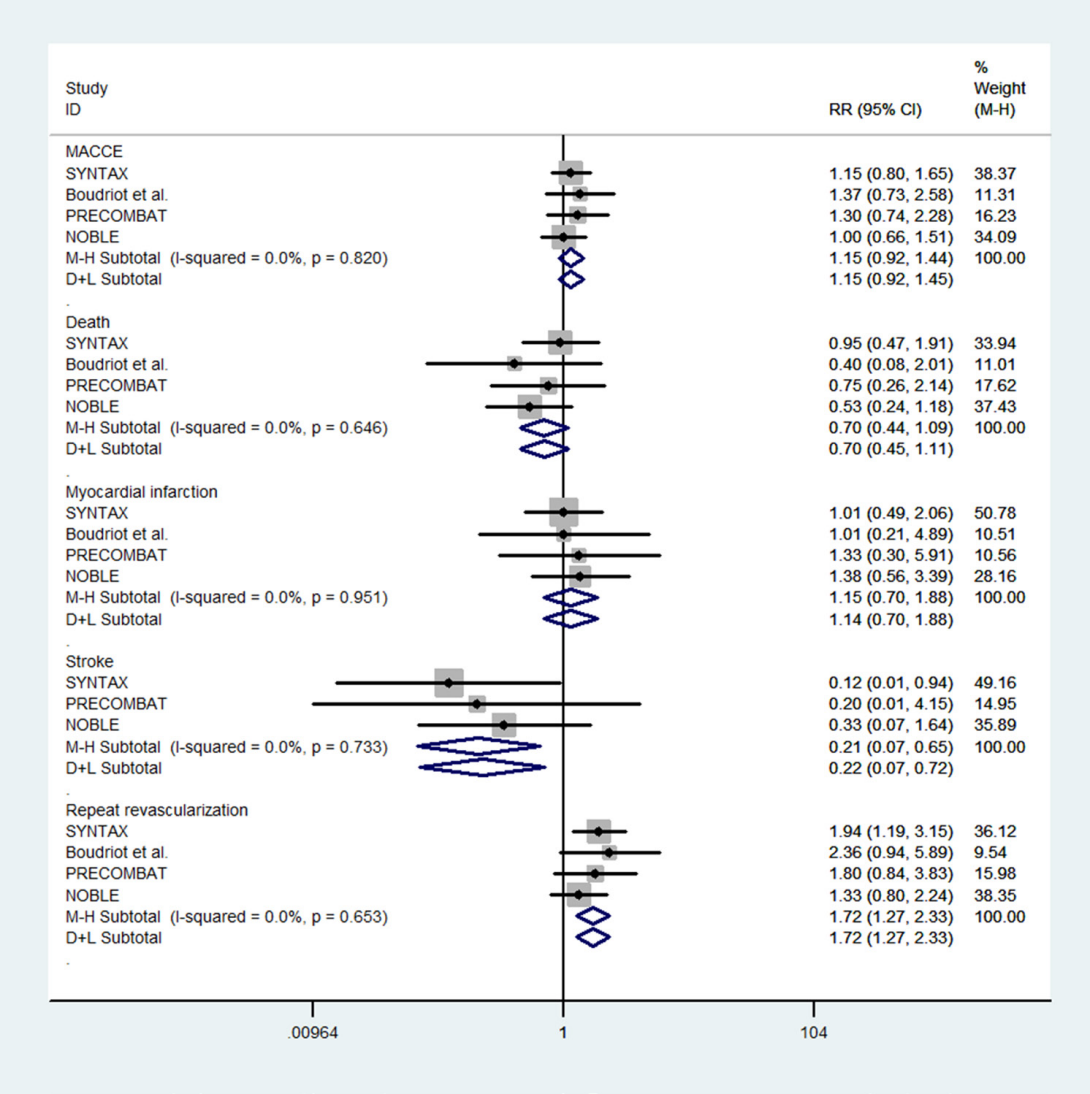
Forest plot for 1-year outcomes of PCI using DES and CABG group

Five trails reported the one-year incidences of MACCE, all-cause death, MI, and RR, and four trails reported the outcome of stroke. Overall, when all the studies were pooled in the meta-analysis, there were no significant differences in one-year outcomes of MACCE, all-cause death, and MI between PCI using DES and CABG for the treatment of LMCA disease (MACCE: RR = 1.15, 95% CI = 0.92–1.44, *P* = 0.232; all-cause death: RR = 0.70, 95% CI = 0.45–1.09, *P* = 0.112; MI: RR = 1.15, 95% CI = 0.70–1.88, *P* = 0.590), with no heterogeneity (MACCE: I^2^ = 0%, *P* = 0.820; all-cause death: I^2^ = 0%, *P* = 0.646; MI: I^2^ = 0%, *P* = 0.951) for the outcomes across the trails (Figure 2 and Table 3). Moreover, the results of our analysis indicated that compared with CABG, PCI using DES can reduce the risk of stroke (RR = 0.21, 95% CI = 0.07–0.65, *P* = 0.007), with no heterogeneity (I^2^ = 0%, *P* = 0.733) across the trails (Figure 2 and Table 3). However, there was a significant trend toward a higher risk of RR with PCI using DES versus CABG (RR = 1.72, 95% CI = 1.28–2.33, *P* < 0.001), with no heterogeneity (I^2^ = 0%, *P* = 0.653) across the trails (Figure 2 and Table 3).

**Table 3:** Summary estimates for 1-year outcomes of PCI using DES and CABG group

Outcome	*N*	Test of association				Heterogeneity analysis			Publication bias	
		RR (95% CI)	*Z*	*P*-value	Model	*Q*-value	*P*	I^2^ (%)	Begg’s *P*	Egger’s *P*
Overall										
MACCE	4	1.15 (0.92–1.44)	1.2	0.232	F	0.92	0.82	0.0	0.308	0.287
Death	4	0.70 (0.45–1.09)	1.59	0.112	F	1.66	0.646	0.0	0.734	0.389
MI	4	1.15 (0.70–1.88)	0.54	0.590	F	0.34	0.951	0.0	1.000	0.714
Stroke	3	0.21 (0.07–0.65)	2.68	0.007	F	0.62	0.733	0.0	1.000	0.656
RR	4	1.72 (1.28–2.33)	3.54	< 0.001	F	1.63	0.653	0.0	0.734	0.515

### Long-term outcomes

**Figure 3:**
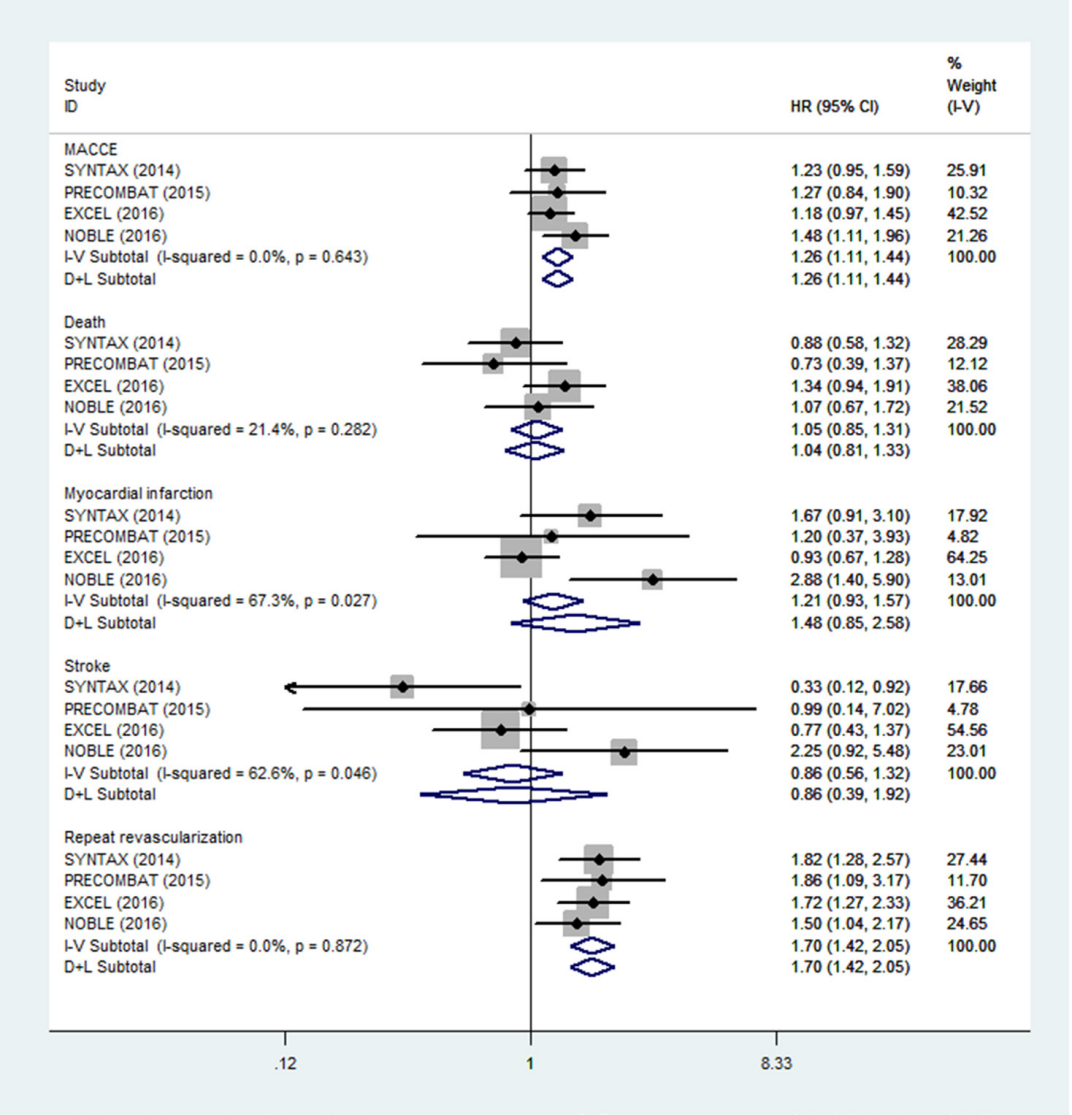
Forest plot for long-term outcomes of PCI using DES and CABG group

Four trails reported the Kaplan-Meier long-term estimates of MACCE, all-cause death, MI, stroke, and RR. There were no significant differences in long-term outcomes of all-cause death, cardiac-death, non-cardiac death, MI, and stroke between PCI using DES and CABG for the treatment of LMCA disease (all-cause death: HR = 1.05, 95% CI = 0.85–1.31, *P* = 0.643; cardiac-death: HR = 1.02, 95% CI = 0.76–1.36, *P* = 0.914; non-cardiac death MI: HR = 1.65, 95% CI = 0.98–2.78, *P* = 0.060; stroke: HR = 0.86, 95% CI = 0.39–1.92, *P* = 0.720), with no significant heterogeneity for all-cause death (I^2^ = 21.4%, *P* = 0.282), cardiac-death (I^2^ = 20.5%, *P* = 0.287), and non-cardiac death (I^2^ = 0.0%, *P* = 0.781), but significant heterogeneity for MI (I^2^ = 67.3%, *P* = 0.027) and stroke (I^2^ = 62.6%, *P* = 0.046) across the trails (Figure 3 and Table 4). However, there were significant trends toward higher risks of MACCE, RR and TVR with PCI using DES versus CABG (MACCE: HR = 1.26, 95% CI = 1.11–1.44, *P* = 0.001; RR: HR = 1.70, 95% CI = 1.42–2.05, *P* < 0.001; TVR: HR = 1.57, 95% CI = 1.25–1.98, *P* < 0.001), with no heterogeneity (MACCE: I^2^ = 0%, *P* = 0.643; RR: I^2^ = 0.0%, *P* = 0.872; TVR: I^2^ = 0.0%, *P* = 0.546) across the trails (Figure 3 and Table 4).

**Table 4:** Summary estimates for long-term outcomes of PCI using DES and CABG group

Outcome	*N*	Test of association				Heterogeneity analysis			Publication bias	
		HR (95% CI)	*Z*	*P*-value	Model	*Q*-value	*P*	I^2^ (%)	Begg’s *P*	Egger’s *P*
Overall										
MACCE	4	1.26 (1.11–1.44)	3.47	0.001	F	1.67	0.643	0.0	0.602	0.949
Death	4	1.05 (0.85–1.31)	3.82	0.643	F	3.82	0.282	21.4	0.174	0.190
Cardiac	4	1.02 (0.76–1.36)	0.11	0.914	F	3.77	0.287	20.5		
Noncardiac	2	1.65 (0.98–2.78)	1.88	0.060	F	0.08	0.781	0.0		
MI	4	1.48 (0.85–2.58)	1.38	0.168	R	9.19	0.027	67.3	0.497	0.446
Stroke	4	0.86 (0.39–1.92)	0.36	0.720	R	8.02	0.046	62.6	1.000	0.988
RR	4	1.70 (1.42–2.05)	5.72	< 0.001	F	0.71	0.872	0.0	0.497	0.707
TVR	3	1.57 (1.25–1.98)	3.81	< 0.001	F	1.21	0.546	0.0		
Early generation DES										
MACCE	2	1.24 (0.99–1.54)	1.95	0.052	F	0.02	0.897	0.0	0.317	-
Death	2	0.83 (0.59–1.17)	1.05	0.295	F	0.24	0.626	0.0	0.317	-
MI	2	1.56 (0.90–2.68)	1.6	0.111	F	0.24	0.626	0.0	0.317	-
Stroke	2	0.42 (0.17–1.03)	1.9	0.058	F	0.95	0.329	0.0	0.317	-
RR	2	1.83 (1.37–2.45)	4.07	< 0.001	F	0.00	0.947	0.0	0.317	-
New generation DES										
MACCE	2	1.27 (1.08–1.50)	2.88	0.004	F	1.63	0.202	38.5	0.317	-
Death	2	1.24 (0.93–1.64)	1.46	0.144	F	0.56	0.455	0.0	0.317	-
MI	2	1.13 (0.84–1.51)	0.78	0.434	R	7.89	0.005	87.3	0.317	-
Stroke	2	0.86 (0.39–1.92)	0.23	0.819	R	3.90	0.048	74.4	0.317	-
RR	2	1.63 (.29–2.06)	4.08	< 0.001	F	0.32	0.872	0.0	0.317	-

### Sensitivity analysis

In sensitivity analyses performed by DES type for long-term outcomes, there remained no difference between PCI using DES and CABG in terms of death, MI, and stroke (Table 4), PCI continued to have a significantly higher risk of RR, regardless of DES type used (Table 4). However, there was higher risk of MACCE with PCI using DES versus CABG only in new-generation DES group (HR = 1.27, 95% CI = 1.08–1.50, *P* = 0.004), and no significant difference in early-generation DES group (HR = 1.24, 95% CI = 0.99–1.54, *P* = 0.052) (Table 4).

### Publication Bias

We constructed funnel plots and carried out Begg’s rank test and Egger’s linear regression test to assess whether publication bias affected the results of the studies. We found no evidence of funnel plot asymmetry across the studies with respect to each outcome (not shown). The results of Begg’s rank test and/or Egger’s linear regression test also showed no publication bias across the studies with respect to each outcome (all *P* = nonsignificant) (Table 3 and Table 4).

## DISCUSSION

To our best of knowledge, this is the largest meta-analysis of including a total of 5 randomized clinical trials with 4595 LMCA patients to systematically clarify whether PCIs using DES was noninferior on the safety and efficacy to CABG for treatment of LMCA disease during short-term and long-term period. The results of this analysis indicated that PCI using DES was associated with a lower risk of stroke, a higher risk of RR than CABG at 1-year follow-up. In addition, there were significant trends toward higher risks of MACCE and RR with PCI using DES versus CABG at long-term follow-up. The results showed that PCI using DES is noninferior to CABG in short term follow-up of patients with LMCA, but CABG is more safety and efficacy than PCI using DES in long-term follow-up. The reason for this might be CABG for long lesion segments could protect against target lesion and proximal de-novo lesion myocardial infarctions.

One previous meta-analysis identified 1611 patients from 4 randomized clinical trials to determine the safety and efficacy of PCI using DES compared with CABG in patients with LMCA disease, and found that PCI using DES was associated with nonsignificantly different 1-year rates of MACCE, death and MI, a lower risk of stroke and a higher risk of RR compared with CABG [22]. Although, our meta-analysis showed the same results with this previous meta-analysis, the limitations of this previous meta-analysis should not be ignored. First, this previous meta-analysis included the randomized clinical trials and the data regarding the 1-year outcomes were categorical, RRs should be performed for summary statistics, but it used Odds ratios (ORs) to represent the effects which might overestimate effect size. Second, this previous meta-analysis did not enroll the latest NOBLE trail which included 1184 patients with LMCA disease [18]. Additionally, several clinical trials included by this previous meta-analysis reported the long-term outcomes of PCI using DES versus CABG in recent years, it is imperative to evaluate the safety and efficacy of PCI using DES compared with CABG in long-term follow-up. A recent meta-analysis by Nerlekar et al. reported that there was no significant difference in the primary safety end point between the PCI using DES and CABG for treatment of LMCA disease in long term follow-up [26]. However, our results indicated that there was significant trend toward higher risk of MACCE with PCI using DES versus CABG in long-term follow-up. The reason of different results between the two meta-analyses may be that this study by Nerlekar et al. extracted the categorical data of long-term outcomes and used the ORs as the summary statistics which may reduce the statistical power. Actually, all of the trails reported the Kaplan-Meier long-term estimates, and pooled HRs could better reflect the long-term effects.

This meta-analysis included 4 trials which reported the long-term outcomes of PCI using DES versus CABG in patients with LMCA disease, two previous trials (SYNTAX and PRECOMBAT trials) used the first-generation drug-eluting stents and showed no significant difference regarding the risk of MACCE between PCI using DES and CABG [20, 21]. However, medical technology and adjunctive pharmacotherapies of PCI and CABG have developed rapidly, and these results may be dated [27, 28]. Two recently large sample size clinical trials (EXCEL and NOBLE trials) compared the long-term outcomes of the current PCI and CABG practice in LMCA disease [18, 19], but the results of the two trials were inconsistent. The NOBLE trail found CABG was better than PCI using DES with respect to Kaplan-Meier estimates of MACCE and revascularisation for treatment of LMCA disease at 5 years, while the EXCEL trail demonstrated that PCI using DES was noninferior to CABG with respect to the primary composite end point of death, stroke or MI at 3 years. Several reasons may explain the disparate results observed in the two trials. First, the baseline, clinical, and procedural characteristics were not the same in the two trials. For example, the rates of diabetes mellitus, acute coronary syndrome, Off-pump CABG and only arterial conduits used, and SYNTAX score were higher in EXCEL trial than those in NOBLE trial. Baseline clinical and procedural risks may affect the long-term outcomes of LMCA disease [29, 30]. Second, the duration of follow-up was 3 years in EXCEL trial, while the follow-up of NOBLE was 5 years. More adverse outcomes might be investigated in PCI using DES than CABG for treatment of LMCA disease with longer duration follow-up.

Some limitations of this meta-analysis should be addressed. First, the analysis included only 5 studies involving 2297 LMCA patients who underwent PCI using DES and 2298 patients who underwent CABG. The small sample size of the analysis is a major weakness that may have decreased its statistical power to properly compare the safety and efficacy of PCI using DES and CABG. Regarding the analyses in which the studies were stratified by sample size and DES type, the sample sizes of the subgroups were too small, which greatly limited our ability to explore effects in these subgroups. Second, the unavailability of individual data in the trials, we could not evaluate the baseline, clinical, and procedural characteristics which may influence clinical outcomes further. Third, this meta-analysis was based on unadjusted estimates. More precise estimates should be performed to account for the effects of various confounders, including age, sex, disease status and postoperative care. Fourth, for long-term follow-up, three trials reported the 5-year outcomes and one trial showed the 3-year outcomes, thus the long-term follow-up durations of the studies were variable and the results could not truly reflect the long-term effect between the two revascularization strategies.

## CONCLUSIONS

In summary, the results of our meta-analysis demonstrated that PCI using DES is noninferior to CABG in short term follow-up of patients with LMCA disease, but CABG is more safety and efficacy than PCI using DES in long-term follow-up. However, additional well-designed studies that are based on larger sample sizes and involve patients of different clinical characteristics are needed to validate these findings.
